# Clinic Examination and Gene Diagnosis for a Birt–Hogg–Dubé Syndrome Family With a Novel *flcn* Frameshift Mutation Causing Nonsense-Mediated mRNA Degradation

**DOI:** 10.1155/humu/7194418

**Published:** 2025-02-03

**Authors:** Yang Xu, Jie Gao, Yang An, Chenxi Zou, Guoqing Ding, Guohua Yang

**Affiliations:** ^1^Department of Respiratory and Critical Care Medicine, The Eighth Medical Center, Chinese PLA General Hospital, Beijing, China; ^2^Department of Pathology, The First Medical Center, Chinese PLA General Hospital, Beijing, China; ^3^Department of Medical Genetics, School of Basic Medical Sciences, Wuhan University, Wuhan, Hubei, China; ^4^Hubei Provincial Key Laboratory of Developmental Originated Disease, Wuhan, Hubei, China

**Keywords:** BHD syndrome, CRISPR/Cas9, *FLCN*, frameshift mutation, NMD

## Abstract

**Background:** Birt–Hogg–Dubé syndrome (BHD) was an autosomal dominant disorder caused by a mutation in the folliculin (*FLCN*) gene and characterized by benign cutaneous fibrofolliculomas in the head and neck, pulmonary cysts, spontaneous pneumothorax, and combined renal tumors.

**Methods:** This study reported a familial case presenting multiple pulmonary bullae, recurrent spontaneous pneumothorax, diffuse cystic lesions in both lungs, and renal cysts. To further clarify the diagnosis, next-generation sequencing (NGS) was performed in conjunction with the clinical diagnostic criteria for Birt–Hogg–Dubé. The eukaryotic recombinant expression vectors of pEGFP-C1-*FLCN* and knock-in *FLCN* mutation by CRISPR/Cas9 were conducted in 293 T and BEAS-2B cell lines. The mRNA and protein expression of the *FLCN* mutation were verified by fluorescence quantitative PCR and Western blot assay. Nonsense-mediated mRNA decay (NMD) assays and immunohistochemical assays were conducted to elucidate the pathogenicity of the mutation and explore potential mechanisms.

**Results:** A unique, novel, unspecified significance *FLCN* mutation NM_144997.7: c.21_22del (p. Cys8 Profs⁣^∗^28) in Exon 4 was detected in both patients. The results demonstrated that the newly identified *FLCN* frameshift mutation significantly decreased *FLCN* mRNA and protein expression. The NMD complex recognized and degraded mRNAs containing a premature termination codon (PTC) in the open reading frame of the *FLCN* frameshift mutation, resulting in haploinsufficiency and ultimately contributing to the manifestation of BHD. Protein expression on the AMP-activated protein kinase (AMPK), Wnt/*β*-catenin, and mammalian target of rapamycin (mTOR) signaling pathways by immunohistochemistry indicated that *FLCN* frameshift mutations were responsible for BHD through the activation of AMPK, Wnt/*β*-catenin, and mTOR signaling pathways.

**Conclusion:** The study demonstrated that a novel *FLCN* frameshift mutation was responsible for the pathogenesis of BHD and preliminarily demonstrated that *FLCN* causes BHD through the AMPK, Wnt/*β*-catenin, and mTOR signaling pathways.

## 1. Introduction

Birt–Hogg–Dubé syndrome (BHD) is an autosomal dominant disorder first reported in 1977, caused by a mutation in the folliculin (*FLCN*) gene on Chromosome 17 and characterized by benign cutaneous fibrofolliculomas in the head and neck, pulmonary cysts, spontaneous pneumothorax, and combined renal tumors [1, 2]. BHD involves the lungs, skin, and kidneys and may present as a single lesion or as a combination of three organs, with a high degree of heterogeneity in clinical presentation [3]. The responsible gene *FLCN* consists of 14 exons, and the coding region covers Exons 4–14 (NCBI Reference Sequence: NM_144997.6) [4]. Several pathogenic germline mutations of this gene have been reported in BHD [5]. These mutations mainly contain small fragments of insertion/deletion, splicing mutations, and nonsense mutations, among which c.1285dup in Exon 11 is the most common mutation in Caucasian and Chinese populations [6, 7].

*FLCN* mutations have been suggested to play an important role in the pathogenesis of Birt–Hogg–Dubé–related lesions. On the one hand, mutations in the *FLCN* gene lead to *FLCN* haploinsufficiency, which results in lung fibroblast dysfunction and affects lung tissue repair capacity [8]. This was related to the fact that haploinsufficiency of *FLCN* affected the expression of proteins and induced fibroblasts to migrate and to synthesize extracellular matrix, such as TGF-*β*1, fibronectin (FN1), and COL1A1, which in turn affects the chemotactic activity and tissue repair capacity of lung tissues [9]. Fibroblasts and macrophages then secrete large amounts of inflammatory factors, inducing inflammation and changing the lung structure. Eventually, pneumothorax is formed due to the destruction of elastic fibers in the lungs [10]. On the other hand, *FLCN* gene mutations lead to the production of *FLCN* truncated protein and loss of function [11]. *FLCN* protein loss of function can affect a variety of metabolic pathways and cellular processes, including modulation of the mammalian target of rapamycin (mTOR) pathway, regulation of PGC1*α* and mitochondrial biogenesis, cell–cell adhesion and RhoA signaling, control of TFE3/TFEB transcriptional activity, amino acid–dependent activation of mTORC1 on lysosomes through Rag GTPases, and regulation of autophagy [2].

This study includes two members from the same family with bilateral lung cystic lesions diagnosed as BHD and whole exome sequencing analysis detected a unique, novel *FLCN* shifter mutation c.21_22del in Exon 4 (p. Cys8 Profs⁣^∗^28). *FLCN* frameshift mutation may generate premature termination codons (PTCs) that result in the interruption of ribosomal translation and subsequent production of shortened and nonfunctional *FLCN* protein. This activates a regulatory mechanism called nonsense-mediated mRNA decay (NMD) that degrades PTC-containing mRNA, reducing further production of shortened proteins [12]. To explore whether the newly identified *FLCN* mutations contribute to BHD through the NMD pathway, we constructed *flcn*-wt and *flcn*-mut plasmids, transfected by liposome, and knocked in the *FLCN* Exon 4 c.21_22del (p. Cys8 Profs⁣^∗^28) mutation by CRISPR/Cas9 with BEAS-2B cells, which determines whether mRNA produced by nonsense mutations is degraded through the NMD pathway.

*FLCN* is an AMP-activated protein kinase (AMPK)–binding partner [13] identified as a tumor suppressor protein responsible for the BHD in humans [14]. *FLCN*, mutated in BHD, and its interacting partner FNIP1 may be involved in energy and/or nutrient sensing through the AMPK and mTOR signaling pathways. To explore the molecular mechanism of *FLCN* mutations leading to BHD, immunohistochemistry was used to examine the expression of key proteins of the AMPK/mTOR signaling pathway.

This work systematically evaluated the clinical phenotype of the *FLCN* deletion out-of-frame mutation, verified whether the mutation leads to the disease through the NMD pathway and the possible molecular mechanisms, and provided theoretical guidance for exploring the mechanism of BHD and the early diagnosis of BHD.

## 2. Materials and Method

### 2.1. Subjects

A patient was admitted to the Chinese PLA General Hospital with unexplained recurrent pneumothorax and multiple diffuse cystic changes in both lungs. Combined with clinical signs and symptoms, there was a strong clinical suspicion that the onset of the disease was related to BHD. And then next-generation sequencing (NGS) identified an *FLCN* variation of uncertain significance. Subsequently, Sanger sequencing of the remaining four members of the entire family resulted in the detection of an *FLCN* mutation at the same locus in one additional member of the family. Genetic diagnosis met with the diagnostic criteria for Birt–Hogg–Dubé due to *FLCN* mutations. All the patients were clinically assessed by at least two experienced clinicians to clarify the diagnosis and understand the genetic background. All participants collected in this study provided informed consent for the study approved by the ethics committee of the Chinese PLA General Hospital. Patients highly suspected of BHD underwent lung CT scanning, renal MRI, and skin examination.

### 2.2. Diagnostic Criteria for BHD

Primary diagnostic criteria were as follows: (1) the presence of more than five fibrofolliculomas or trichiliocosm in adults, of which at least one has been confirmed pathologically, and (2) the presence of a germline mutation in the *FLCN* gene that causes the disease.

Secondary diagnostic criteria were as follows: (1) multiple pulmonary cysts, bilateral occurrence of lesions located at the base of the lungs with no other clear etiology, with or without a history of primary spontaneous pneumothorax; (2) renal tumors: relatively early age of onset (< 50 years old), bilateral or multiple renal tumors, or pathological type of mixed acidophilic cell carcinoma and pheochromocytoma; and (3) confirmed diagnosis of BHD in a first-degree relative. The diagnosis of BHD was made by fulfilling one major or two minor criteria.

### 2.3. Whole-Exome Sequencing Analysis

The genomic DNA was analyzed by target region capture high-throughput sequencing. The assay was performed on the Illumina sequencing platform. The data obtained were sequenced at an average depth of 90× for known exons and upstream and downstream 5 bp sequences in the human genome. The average sequencing depth of the sequences was 90×, and 98% of the target sequences were sequenced at a depth of 20× or more. Base identification was performed for all sequenced segments. The assay was developed and validated by metallic medicine. Sequencing data were analyzed using the GATK software package. The sequenced segments were compared with the UCSC hg19 reference gene group by BWA (Burrows–Wheeler Aligner). The sequenced segments were compared with the UCSC hg19 reference gene group by BWA. The test uses VEP (Variant Effect Predictor) software to annotate variants. The test was also based on genetic disease databases such as ClinVar, OMIM, HGMD, and gnomAD; variant databases; and human population large-scale sequencing databases. Variants were screened based on genetic disease databases such as ClinVar, OMIM, HGMD, and gnomAD; variant databases; and human-scale sequencing databases.

### 2.4. Construction of *FLCN*-wt and *FLCN*-mut Expression Vectors

The whole gene-synthesized *FLCN* CDS (coding sequence) was used as the template, and Phage-*FLCN*-BamHI-F/Phage-*FLCN*-NotI-R and Phage-*FLCN*-BamHI-mut-F/Phage-*FLCN*-NotI-R were used as the primers to amplify to obtain the fragments of BamHI-wt-NotI and BamHI-mut-NotI, which were inserted into the phage vector after double enzyme digestion of BamHI and NotI, and then the Phage-wt and Phage-mut vectors were obtained, and the sequence verification was performed. The primer sequence for vector construction is listed in Table 1.

### 2.5. Cell Culture and Transfection

The 293 T and BEAS-2B cells purchased from Priscilla (Wuhan, Hubei Province, China) were cultured with DMEM (Gbico, Carlsbad, CA, United States) medium containing 10% fetal bovine serum (FBS) (Yeasen, Shanghai, China) and 0.5% penicillin/streptomycin (Yeasen, Shanghai, China). The cells were grown in six-well plates until they reached 70%–80% confluency, and the constructed recombinant expression vectors were transfected into the 293 T cells or BEAS-2B cells instantaneously using Opti-MEM (Lonza, Bassel, Switzerland) and Lipofectamine 3000 (Carlsbad, CA, United States) transfection reagent. After transfection for 48 h, the samples were collected for qPCR and Western blot (WB) detection, respectively.

### 2.6. NMD Detection by Cell Transfection of *FLCN* Mutation

#### 2.6.1. Cycloheximide (CHX) Treatment

The eukaryotic recombinant expression vectors of pEGFP-C1-*FLCN* wt and mut types validated for gene expression were transiently transfected into 293 T cells for 40 h, followed by the addition of CHX (500 *μ*M) (Aladdin, Shanghai, China) treatment for 8 h, and then subjected to qPCR assay.

#### 2.6.2. si-UPF1 Treatment

The small interfering RNA si-UPF1 was constructed and the target sequence was 5⁣′-GACTCTGGTAATGAGGATTTA-3⁣′. The eukaryotic recombinant expression vectors of pEGFP-C1-*FLCN* wt and mut types, which were validated for gene expression, were cotransfected with small interfering RNA si-UPF1 (50 nmol/mL) into 293 T cells, respectively, and the cell samples were collected for qPCR and WB assays after 48 h. The primer sequence for RT-qPCR is listed in Table 2.

### 2.7. NMD Detection by Cells Knock-in *FLCN* Mutation With CRISPR/Cas9

#### 2.7.1. The Design of sgRNA and Construction of sgRNA Expression Vector

According to the mutation information, the editing site was determined, and the exon sequence of 23–250 bp was selected for the software input box of the online sgRNA design (http://crispr.mit.edu/). Then the design calculation was carried out, and the sgRNA sequence would be automatically output by the software. A pair of complementary DNA oligo sequences were synthesized by selecting sgRNA template sequences with a low miss probability. The target sequence and primer sequence are shown in Table 3. Then, we obtain the target fragment by double-chain annealing. The double-chain annealing system and procedure are as follows. The PCR reaction mixture consisted of 2.5 *μ*L of 2× mix, 1 *μ*L of Primer-F, 1 *μ*L of Primer-R, 1 *μ*L of gDNA (mut), and 24.5 *μ*L of double distilled water (ddH_2_O). The reaction temperature is 57°C. Then, the expression vector containing the target gene was obtained by linking and transforming the target fragment with the Px459 vector enzymatically cut at the BbsI site, and then sequencing was conducted for identification.

#### 2.7.2. sgRNA Target Validation

The sgRNA plasmids were transfected into BEAS-2B cells. After 48 h, 2 *μ*g/mL puromycin (Beyotime, Shanghai, China) was added to screen positive cells until about 60% of the cells died. gDNA was extracted from the cells for PCR amplification, and the PCR products were connected to the T vector for sequencing verification.

#### 2.7.3. Donor Vector Construction

Two pairs of nested primers, 8212-*FLCN*-F and 9904-*FLCN*-R, 8537-*FLCN*-F, and 9592-*FLCN*-R, were designed for nested PCR using normal human DNA. The second-round amplification product of nested PCR was used as the template. 8537-*FLCN*-F and *FLCN*-mut-R were used as the primers to obtain the mutated Fragment 1 of 1530 bp, and *FLCN*-mut-F and 9592-*FLCN*-F were used as the primers to obtain the mutated Fragment 2 of 2554 bp. Fragment 1 and Fragment 2 were purified and mixed as a template according to 1:1. 8537-*FLCN*-F and 9592-*FLCN*-R were used as the primers to obtain the donor fragment of 1054 bp. The donor fragment of 1054 bp was purified and connected to the T vector, and then the donor vector was obtained. The primer sequence is shown in Table 4.

#### 2.7.4. Screening and Identification of Single-Cell Clones

The sgRNA plasmid was cotransfected with the donor plasmid (sgRNA plasmid: donor plasmid = 3:1), and 2 *μ*g/mL puromycin was added to screen for positive clones after transfection for 48 h. One hundred to two hundred positive cells were added to a 100 mm petri dish and cultured for 10–15 days until a monoclonal was formed. The monoclines were selected and transferred to 48-well plates for culture, part of the cells was collected for sequencing verification, and the remaining cells were passed to 12-well plates for seed preservation according to the sequencing results.

#### 2.7.5. CHX and si-UPF1 Treatment

*FLCN*-KI cells in the control group and the editing group were, respectively, added with CHX (500 *μ*M) (Aladdin, Shanghai, China) for 8 h. Cell was collected, and the total RNA was routinely extracted by TRIzol and then reversed into cDNA. The expression levels of target genes in the control group and editing group were detected by RT-qPCR.

The small interfering RNA si-UPF1 was constructed, and the target sequence was 5⁣′-GACTCTGGTAATGAGGATTTA-3⁣′. The si-UPF1 (50 nmol/mL) was transferred to *FLCN*-KI cells of the control group and the editing group, respectively. The cells were collected after transfection at 48 h, and the total RNA was routinely extracted by TRIzol and then reversed into cDNA. The expression levels of target genes in the control group and editing group were detected by RT-qPCR.

### 2.8. RT-PCR

Cell samples were collected after transfection with wild and mutant eukaryotic recombinant expression vectors for 48 h. Total RNA was extracted by the TRIzol (TaKaRa, Otsu, Shiga, Japan) method, and cDNA was synthesized after DNA digestion. The expression levels of *FLCN* were detected by qPCR.

Eukaryotic recombinant expression vectors of wt and mut were transfected into 293 T cells, and then cell samples were collected after CHX treatment or RNA si-UPF1 interference. Total RNA was routinely extracted using the TRIzol method, and cDNA synthesis was performed after DNA removal by digestion. The expression levels of UPF1 and *FLCN* were detected by qPCR.

*FLCN*-KI cells were collected from the control and editing groups, and the total RNA was extracted by TRIzol and then reversed into cDNA. The expression levels of target genes in the control group and editing group were detected by RT-qPCR. The primer sequence for RT-qPCR is listed in Table 5.

### 2.9. WB

Cell precipitation was collected after transfection with eukaryotic recombinant expression vectors for 48 h. The total protein was extracted by radioimmunoprecipitation assay (RIPA) lysate (containing protease/phosphatase inhibitors), the protein concentration was determined by the bovine serum albumin (BSA) kit, and then the protein was denatured. The same amount of total protein was taken for sodium dodecyl polyacrylamide gel (SDS-PAGE) electrophoresis, and the expression levels of wild-type and mutant target proteins were detected by WB.

### 2.10. Immunohistochemistry

Both one Birt–Hogg–Dubé patient and 15 non-Birt–Hogg–Dubé patients were obtained from the department of pathology of Chinese PLA General Hospital. Specimens of lung tissue of Birt–Hogg–Dubé patients were obtained from the proband, and non-Birt–Hogg–Dubé patients with pulmonary herniation were from patients with lung CT suggestive of typical pulmonary herniation, did not meet the clinical diagnostic criteria for Birt–Hogg–Dubé, and were pathologically diagnosed as simple pulmonary bulla. All lung tissues were fixed in 4% paraformaldehyde and made into 5 *μ*m paraffin sections. Paraffin-embedded tissues were incubated at 72°C prior to dewaxing and rehydration. Antigen retrieval was achieved by placing sections in 0.01 M citric acid (pH 6) and microwaving for 15 min. Endogenous peroxidases were quenched in 15 mL of 3% hydrogen peroxide. Samples were washed again with phosphate-buffered saline (PBS) prior to treatment with 10% BSA blocking solution and incubated at 37°C for 40 min. Tissues were incubated in mouse anti-human *FLCN*, p-mTOR, p-SMAD3, and *β*-catenin monoclonal antibodies. The primary antibodies were diluted in TBST (tris buffer solution tween) at 4°C overnight. Samples were washed with PBS on the following day, incubated in a secondary antibody (1:1000), washed, and then treated using the 3,3⁣′-diaminobenzidine (DAB) peroxidase staining kit (Shanghai Biotechnology Company, NO132, Xuhui District, Shanghai City, China) as per the manufacturer's protocol. For detection, a DAB peroxidase detection kit was used, and color development was monitored using an optical microscope. The development process was terminated by removing DAB and rinsing the sections with ddH_2_O for 1 min prior to counterstaining with hematoxylin.

The intensity of immunohistochemical staining of sections of the same specimen was determined by two senior pathologists in a double-blind manner, and if the results were not uniform, the final intensity of staining was determined by repeated reading of the sections by agreement.

### 2.11. Statistical Analysis

All data were expressed as the mean ± SD (standard deviation), calculated using GraphPad Prism 9.0 software (La Jolla, CA, United States) through one-way analysis of variance (ANOVA), followed by the Dunnett test. The sequencing data was analyzed using SnapGene software. *p* values were represented in figures as follows: ⁣^∗^*p* < 0.05, ⁣^∗∗^*p* < 0.01, and ⁣^∗∗∗^*p* < 0.001 were considered significant. Data are shown in column bars representing the mean ± SEM of at least three independent experiments.

## 3. Results

### 3.1. The Process of Confirming the Diagnosis of Birt–Hogg–Dubé Patients

The patient was a 50-year-old male with multiple pulmonary cysts in both lungs since 2018 and a history of intermittent pneumothorax. The patient's health check in August 2018 showed diffuse cystic changes in both lungs on CT examination without chest tightness and breathlessness, cough, and chest pain, and no special treatment was given. In May 2020, the patient had a sudden onset of right-sided chest pain with severe dyspnea after strenuous activity. Lung CT showed that the right pneumothorax with a compression area greater than 50% had diffuse cystic changes in both lungs. Closed chest drainage was given on an emergency basis, and the patient's symptoms resolved. In August 2021, the patient again developed respiratory distress after being active, and the lung CT indicated right-sided pneumothorax with right-sided compressive atelectasis and diffuse cystic changes in both lungs. He was admitted to the hospital for further treatment.

History of past illness shows appendicitis surgery at Age 11 and closed chest drainage for spontaneous pneumothorax in 2018. The patients have lived in Beijing for a long time, with no history of living in infected areas, epidemics, or epidemic water; no history of living in pastoral areas, mines, high-fluoride areas, or low-iodine areas; no history of exposure to chemical substances, radioactive substances, or toxic substances; and no history of drug exposure. But the proband had a history of smoking for 10 years, about 20 cigarettes/day, and occasional alcohol consumption. He has an age-appropriate marriage, with his spouse and son healthy. His mother had a history of recurrent pneumothorax and died of cor pulmonale.

**Figure 1:**
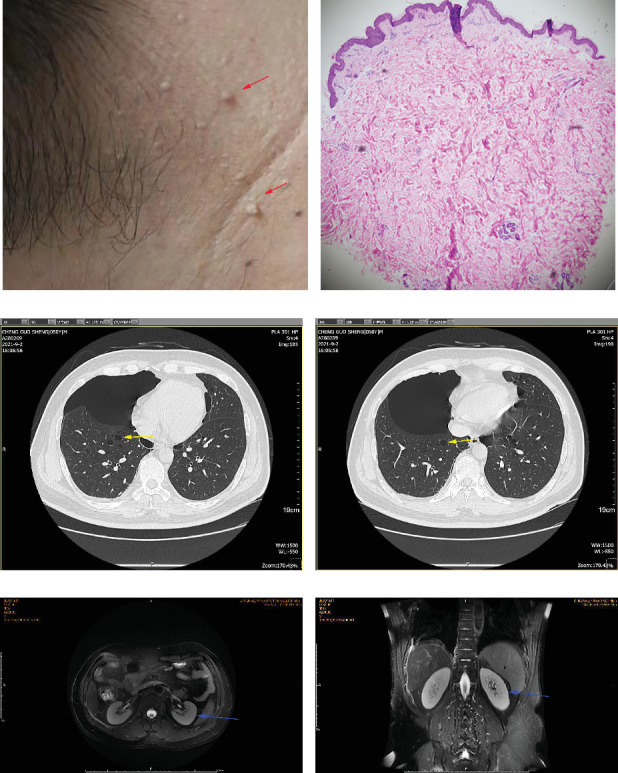
Papules pathological examination, chest CT, and renal MRI results of proband. (a) Papules on the back of the neck. Red arrows indicate papules. (b) Pathological examination results of the papules (HE staining, 40×). Characteristics consistent with fibrofolliculomas. (c, d) Multiple diffuse cystic changes in both lungs. Chest CT showed right pneumothorax, compression area greater than 40%, multiple diffuse cystic changes in both lungs, thin wall, irregular shape, uneven size, located in both lower lungs and mediastinum. Yellow arrows indicate lung cystic. (e, f) Renal magnetic resonance plain scan and dynamic enhancement of the left renal cyst indicated a long T2 signal shadow with a clear punctate boundary in the left kidney (e) and the lesion signal did not decrease in the reverse phase image (f). No abnormal enhancement was found in dynamic enhanced scan. Blue arrows indicate renal cyst.

Physical examination is as follows: decreased breath sounds in the right lung, no dry moist rales were heard in both lungs, and regular heart rate. Multiple small papules were seen on the posterior neck (Figure 1(a)). Pathologic biopsy is as follows: skin grayish-white tissue, size 0.4 × 0.3 × 0.2 cm. Chronic inflammation of squamous epithelial mucosa, epidermal hyperplasia with hyperkeratosis, and significant proliferation of collagenous fibrous tissue seen in the dermis (Figure 1(b)), characteristics consistent with fibrofolliculomas. Pathological examination indicates dermatofibroma, known as acrochordon. Chest CT showed right pneumothorax and multiple diffuse cystic changes in both lungs (Figures 1(c) and 1(d)). Renal magnetic resonance plain scan and dynamic enhancement of renal indicated that the left renal cyst and dynamic enhancement scans showed no signs of abnormal enhancement. No obvious abnormality was found in the right kidney (Figures 1(e) and 1(f)).

**Figure 2:**
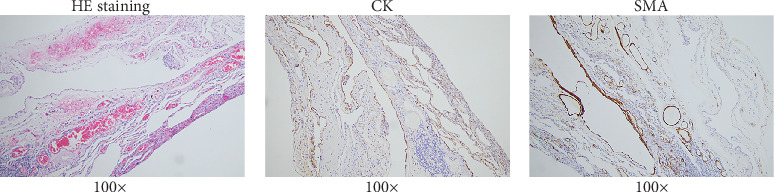
Postoperative pathological of pulmonary alveoli. (a) Pulmonary alveolar pathology results (100×). (b) Immunohistochemistry results of CK in pulmonary alveolar. (c) Immunohistochemistry results of SMA in pulmonary alveolar.

The patient underwent thoracoscopic right lung bulla resection in the department of thoracic surgery in September 2021 because of multiple pulmonary cysts and recurrent spontaneous pneumothorax. Multiple pulmonary cysts were seen in the upper and lower lobes of the right lung, which were excised in stages with the Endo Gia Ultra cutting suture and ligated with multiple pulmonary cysts, respectively. The wedge resection of lung tissue postoperative pathology is as follows: right lung wedge resection tissue was off-white, with several cysts. Bullosa formation was observed under the pulmonary membrane, fibrous capsular wall tissue was observed, foam cell aggregation and multinuclear giant cell reaction were observed within it, and vascular dilation and congestion were evident in the surrounding lung tissue (Figure 2(a)). Immunohistochemical analysis of wedge resection of lung tissue illustrated positive cytokeratin (CK) and positive smooth muscle actin (SMA) (Figures 2(b) and 2(c)).

### 3.2. Genealogical Analysis of Birt–Hogg–Dubé Patients

The proband's CT examination showed diffuse cystic changes in both lungs, The proband's renal magnetic resonance plain scan and dynamic enhancement of renal indicated that left renal cyst. The proband was highly suspected of Birt–Hogg–Dubé, and whole exome sequencing was performed to verify the causes of the disease. Gene test revealed a unique deletion mutation in Exon 4 of the *FLCN* gene (*FLCN* NM_144997.7:c.21_22del p. Cys8Profs⁣^∗^28) among the mutations associated with the disease phenotype, which was not embodied in the Leiden Open Variant Database and was not reported in the literature.

**Figure 3:**
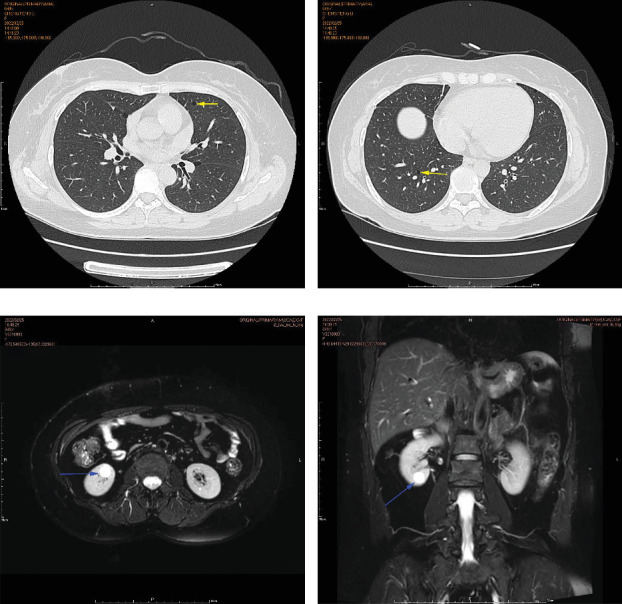
Chest CT and renal MRI results of proband's Sister 1. (a, b) Multiple diffuse cystic changes in both lungs. Chest CT showed multiple diffuse cystic changes in both lungs, thin wall, irregular shape, and uneven size, located in both lower lungs and mediastinum. Yellow arrows indicate lung cystic. (c, d) Renal magnetic resonance plain scan and dynamic enhancement of the left renal cyst indicated a long T2 signal shadow with a clear punctate boundary in the left kidney (c) and the lesion signal did not decrease in the reverse phase image (d). No abnormal enhancement was found in the dynamic enhanced scan. Blue arrows indicate renal cyst.

Chest CT of the proband's Sister 1 of the prognosticator suggests multiple pulmonary cysts in both lungs in January 2022 (Figures 1(d) and 3(a)). The renal MRI of the proband's Sister 1 showed that renal cysts of about 15–17 mm in diameter were at the lower pole of the right kidney and the upper level of the left kidney, with no abnormal enhancement on dynamic enhancement scanning (Figures 1(f) and 3(d)). Proband's Sister 1 gene test also detected mutations in the same locus of the *FLCN* gene. The proband's mother has already passed away, so genetic testing was not conducted. Based on her history of recurrent pneumothorax and death due to cor pulmonale, it was speculated that the mutation was inherited from the mother.

**Figure 4:**
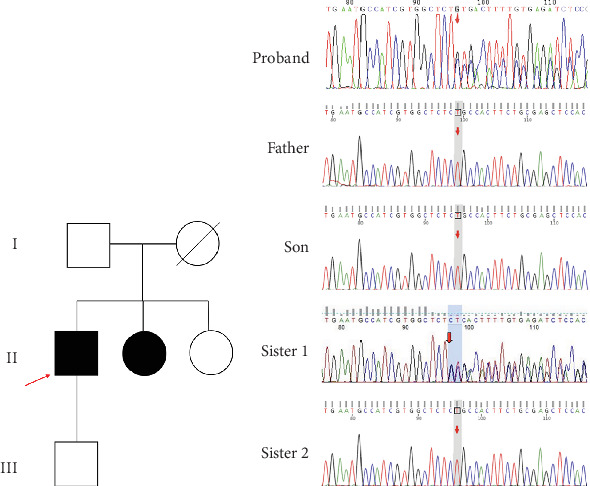
Family pedigree and sequence electropherograms showing the *FLCN* gene mutation in a 50-year-old man and his family members. (a) Pedigree illustrating the segregation of the mutant alleles to the index patient. The proband is indicated by an arrow. Solid symbols represent family members with clinically manifested disease. Hollow symbols represent family members without clinical manifestations. The proband's mother has passed away. (b) Sequence analysis of the *FLCN* gene revealed two heterozygous mutations for c.21_22del (p. Cys8 Profs⁣^∗^28).

No significant abnormalities were seen on CT and MRI of the lungs in other members of the family. *FLCN* gene tested as wild type in the proband's father, the proband's Sister 2, and the proband's son (Figures 4(a) and 4(b)).

### 3.3. The mRNA and Protein Levels of *FLCN* Were Significantly Downregulated by Transfection of *FLCN* Mutation

**Figure 5:**
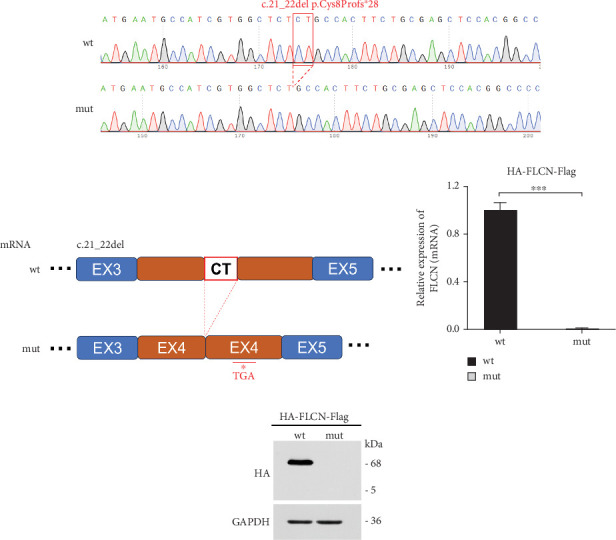
(a) *FLCN*-wt/mut sequencing results showed that mutation c.21_22del p. Cys8 Profs⁣^∗^28 was successfully constructed. (b) Frameshift and nonsense mutations occurring on mutant *FLCN*. (c) Relative mRNA level of wt and mut *FLCN.* (d) Western blot analysis of HA-*FLCN*-flag-wt or HA-*FLCN*-flag-mut (HA-tagged). ⁣^∗^ indicates *p* value less than 0.05. ⁣^∗∗∗^*p* < 0.001.

*FLCN* mutation c.21_22del p. Cys8Profs⁣^∗^28 results in the generation of the PTC in Exon 4 (Figure 5(a)). Bioinformatics analysis suggested that the deletion of bases at Positions 21 and 22 in *FLCN* resulted in a frameshift mutation and the generation of PTC, leading to the generation of the truncated *FLCN* protein (Figure 5(b)). HA-*FLCN* was adopted, which was used to measure the relative mRNA levels and protein expression of wild-type and mutant *FLCN*, respectively. qPCR was performed to determine the relative mRNA levels. In comparison to the wild-type group, the relative mRNA levels in the mutant group decreased significantly (Figure 5(c)). Subsequently, the expression of corresponding proteins was detected by means of WB. Because of the presence of PTC, the mut group would generate truncated proteins. In Phage-HA-*FLCN*-flag, the mutant protein was significantly decreased, suggesting that the truncated protein may express in very low amounts due to premature termination (Figure 5(d)). Taken together, these data suggested that leads to a decrease in mRNA-expressing levels and lower protein expression. These effects were likely partially responsible for the loss of *FLCN* protein function in this Birt–Hogg–Dubé patient.

### 3.4. Transfected *FLCN* Mutation Reduced *FLCN* mRNA Expression Through the NMD System

**Figure 6:**
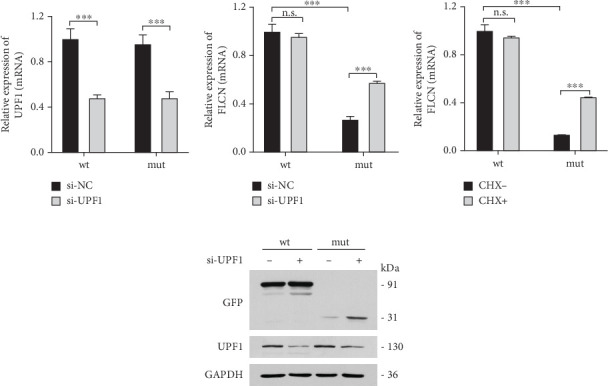
(a) UPF1 siRNA treatment significantly decreased the mRNA level of UPF1. (b) UPF1 siRNA treatment significantly increased the mRNA level of mutant *FLCN*. (c) CHX treatment increased the mRNA level of mutant *FLCN* but not that of wild-type *FLCN*. (d) UPF1 siRNA treatment significantly increased the protein level of mutant *FLCN* but not that of wild-type *FLCN*. ⁣^∗∗∗^*p* < 0.001.

It was hypothesized that 5⁣′ proximal PTCs circumvent NMD by reinitiating translation downstream [15]. *FLCN* mutations c.21_22del p. Cys8Profs⁣^∗^28 reported in this study were located too close to the initiation codon and therefore may escape NMD, not causing changes in mRNA expression levels. Bioinformatics prediction, NMD assay, PCR, and WB were used to verify whether *FLCN* mutations affected protein expression and led to the clinical phenotype through the NMD pathway. The online bioinformatics prediction (https://nmdpredictions.shinyapps.io/shiny) results (Figure S1) also suggested that the mutation site was in the NMD-competent region where NMD may occur. The degradation of mRNAs containing premature stop codons has been attributed to NMD, which may explain the observed decrease in mutant *FLCN* mRNA levels compared to wild type. To investigate this hypothesis, we initially suppressed the expression of UPF1, a crucial constituent of the NMD pathway, and subsequently assessed its impact on the *FLCN* mRNA abundance. In comparison to the si-NC group, the si-UPF1 group exhibited a significant downregulation in both mRNA levels (Figure 6(a)) and protein levels (Figure 6(d)) of UPF1 in cells of wild type and mutant type. However, the knockdown of UPF1 expression resulted in a significant increase in mutant *FLCN* mRNA and protein levels, while the levels of wild-type *FLCN* remained unaffected (Figures 6(b) and 6(d)).

To investigate the impact of NMD, wild-type or mutant *FLCN* was introduced into 293 T cells and subsequently exposed the cells to CHX, a protein synthesis inhibitor, in order to evaluate its potential to impede NMD-mediated mRNA degradation [16]. In comparison to the wild-type (CHX−) group, the expression of *FLCN* mRNA was notably reduced in the mutant (CHX−) group. The administration of CHX+ did not significantly impact *FLCN* mRNA expression in the wild-type group but effectively reversed the downregulation of *FLCN* mRNA expression in the mutant group (Figure 6(c)). This suggested that CHX prevents the degradation of mutant *FLCN* mRNA through NMD. These findings provided further support for our hypothesis that NMD plays a role in the decreased expression of mutant *FLCN*.

### 3.5. Knock-in *FLCN* Mutation by CRISPR/Cas9 Reduced *FLCN* mRNA Expression Through the NMD System

Here, an efficient strategy to knock-in *FLCN* mutation (c.21_22del p. Cys8Profs⁣^∗^28) into the genomic locus was applied to human bronchial epithelial cell BEAS-2B. The online design software was used to design and select sgRNA site sequences with low miss efficiency (Figure S2(A)). The results of sequencing verification showed that all three targets were successfully edited, and the editing efficiency of Tg1 was the highest (Figure S2(B)). The expression level of *FLCN* mRNA in the mock group and *FLCN*-KI group was detected using primer *FLCN*-qPCR-F/R. The results showed that compared with the mock control, the expression of *FLCN*-KI cell lines decreased to 52% (Figure S2(C)).

The monoclonal cell lines (Figure S3(A)) were sequenced, and the mutant cell lines were identified for culture (Figure S3(B)). According to the sequencing results, Tg1-*FLCN* was preliminarily identified as a positive clone. PCR products were connected to the T vector and transformed into DH5*α*, and monoclonal was selected for sequencing. The sequencing results showed that one DNA strand of Tg1-*FLCN* was successfully introduced with mutation c.21_22del p. Cys8Profs⁣^∗^28, while the other DNA strand was wt type. *FLCN*-KI cell lines were successfully constructed in line with heterozygous mutation requirements (Figure S3(C)).

**Figure 7:**
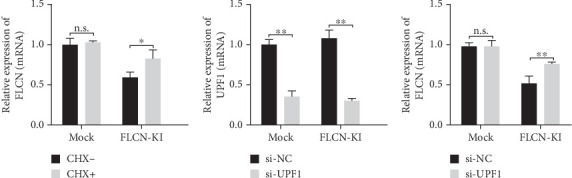
Detection of *FLCN* and UPF1 mRNA expression by RT-qPCR. (a) Relative expression of *FLCN* wild type and mutant mRNA before and after CHX treatment. (b) Inhibition of UPF1 expression in wild and mutant groups after interference with UPF1. (c) The relative expression of *FLCN* wild type and mutant mRNA before and after UPF1 inhibition. Differences between the treated and control groups were marked with ⁣^∗∗^ (*p* < 0.01) to indicate significance. ⁣^∗^ indicates *p* value less than 0.05. ⁣^∗∗^ indicates *p* value less than 0.01.

The expression level of *FLCN* in CHX and RNA si-UPF1-treated mock group and *FLCN*-KI group samples was detected using primers *FLCN*-qPCR-F/R. The expression level of UPF1 in RNA si-UPF1-treated mock group and *FLCN*-KI group samples was detected using primers UPF1-qPCR-F/R. Compared with the mock (CHX−) group, *FLCN* mRNA expression in the *FLCN*-KI (CHX−) group was significantly downregulated. CHX+ treatment had no significant effect on *FLCN* mRNA expression in the mock group. However, the downregulation of *FLCN* mRNA expression in the *FLCN*-KI group was significantly reversed (Figure 7(a)). Compared with the si-NC group, the expression of UPF1 in mock and *FLCN*-KI cells of the si-UPF1 group was significantly downregulated (Figure 7(b)). Compared with the mock (si-NC) group, *FLCN* mRNA expression in the *FLCN*-KI (si-NC) group was significantly downregulated. si-UPF1 had no significant effect on *FLCN* mRNA expression in the mock group but could significantly reverse the downregulation of *FLCN* mRNA expression in the *FLCN*-KI group (Figure 7(c)).

### 3.6. *FLCN* Mutations Lead to the Occurrence of Birt–Hogg–Dubé Through the AMPK, Wnt/*β*-Catenin, and mTOR Signaling Pathways

**Figure 8:**
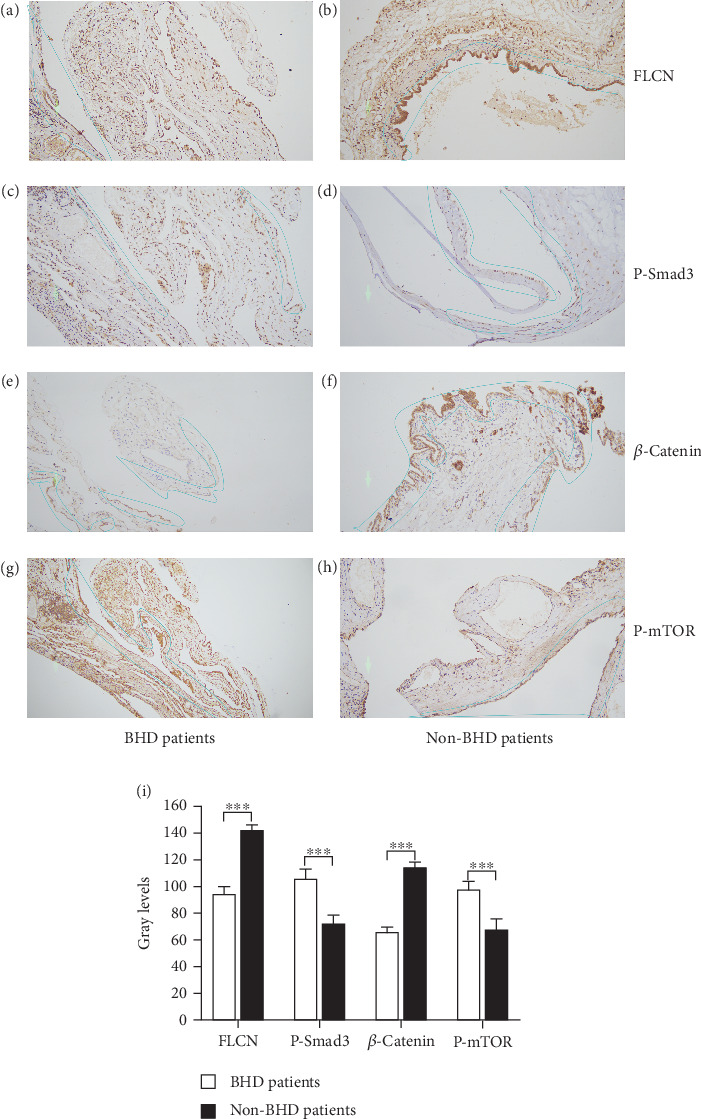
Immunohistochemistry of *FLCN*, P-Smad3, *β*-catenin, and P-mTOR in pulmonary alveolar. Different protein expression levels in BHD patients and simple pulmonary bulla patients: (a, b) *FLCN*, (c, d) P-Smad3, (e, f) *β*-catenin, and (g, h) P-mTOR. (i) Gray levels statistics of *FLCN*, P-Smad3, *β*-catenin, and P-mTOR. The blue area was the gray contrast value area between BHD patients and non-BHD patients.

According to the American College of Medical Genetics and Genomics (ACMG) guidelines for classification criteria, to seek additional pathogenic evidence for *FLCN* mutations, the impacts of *FLCN* on the expression levels of key proteins in signaling pathways in both Birt–Hogg–Dubé and simple pulmonary bulla patients were investigated. Positive expression of *FLCN* in epithelial and mesenchymal cells was seen in both Birt–Hogg–Dubé and simple pulmonary bulla patients, and the grayscale value of positive cells of *FLCN* in Birt–Hogg–Dubé patients was lower than in patients with simple pulmonary bulla (95.000 ± 1.546 vs. 143.067 ± 1.115, Figures 8(a) and 8(b)).

Since the *FLCN* gene was involved in the synthesis of *FNIP-1* as well as *FNIP-2*, where *FNIP-1* was involved in the AMPK pathway [17], FNIP-2 acts on the mTOR pathway and regulates cell growth and reproduction [18]. And the loss of FLCN inhibited canonical Wnt signaling via TFE3 [19]. The expression of AMPK (P-Smad3), Wnt/*β*-catenin (*β*-catenin), and mTOR (p-mTOR) signaling pathways was verified by immunohistochemistry.

The grayscale value of positive cells of P-Smad3 (AMPK signaling pathway) in Birt–Hogg–Dubé patients was higher than in patients with simple pulmonary bulla (107.133 ± 1.759 vs. 72.933 ± 6.017, Figures 8(c) and 8(d)). The grayscale value of positive cells of *β*-catenin (mTOR signaling pathway) in Birt–Hogg–Dubé patients was lower than in patients with simple pulmonary bulla (66.467 ± 1.142 vs. 114.933 ± 1.307, Figures 8(e) and 8(f)). The grayscale value of positive cells of p-mTOR (mTOR signaling pathway) in Birt–Hogg–Dubé patients was lower than in patients with simple pulmonary bulla (98.000 ± 1.630 vs. 67.800 ± 2.209, Figures 8(g) and 8(h)). The gray value differences in *FLCN*, P-Smad3, *β*-catenin, and p-mTOR between Birt–Hogg–Dubé patients and non-Birt–Hogg–Dubé patients were statistically significant (*p* < 0.001, Figure 8(i)).

## 4. Discussion

BHD is characterized by skin lesions in the head and neck, lung lesions, and combined renal tumors [1]. Skin lesions in patients with BHD present as fibrofolliculomas, mainly on the face and neck, as multiple, yellowish or white, papules protruding from the skin surface. Dermal histopathology of the dermis reveals cystic dilated structures of keratinous debris. Trichodiscoma and acrochordons are also visible types of lesions and may be a form of fibrofolliculoma that has progressed to an advanced stage [20].

Lung lesions in patients with BHD present as bilateral, multiple cystic changes, usually thin-walled, irregularly shaped, and heterogeneous in size, located in both lower lungs and near the mediastinum parietal. Patients with Birt–Hogg–Dubé are at risk of spontaneous pneumothorax, with the risk of occurrence 50 times higher than in the normal population, and some patients have recurrent episodes of pneumothorax [21].

Most renal tumors in BHD are bilateral and multiple, and histological types include chromophobe cell carcinoma, clear cell carcinoma, and papillary renal cell carcinoma. The risk of kidney tumors in patients with BHD is seven times higher than in the normal population. The patients lacked the classic symptoms, and only a minority developed the “kidney cancer triad” of lower back pain, hematuria, and abdominal mass [22].

There are ethnic differences in the clinical presentation of Birt–Hogg–Dubé. In Asian Birt–Hogg–Dubé patients, the predominant manifestation is pulmonary cystic lesions (95%) [23], and in European and American patients, the predominant manifestations were skin lesions (90%) and bilateral lung cysts (70%–84%) [24]. The clinical features of Asian Birt–Hogg–Dubé patients were mainly pulmonary cysts and pneumothorax, while skin lesions and renal tumors were less common, and skin lesions were more common in European and American Birt–Hogg–Dubé patients [25]. In China, BHD with an isolated pulmonary phenotype is the most common [21].

In this study, a unique, novel *FLCN* frameshift mutation, highly suspected to be associated with BHD, was reported. A proband with pulmonary cysts and pneumothorax as the main clinical features, without hair follicle hamartomas and kidney tumors. That was specifically consistent with the feature of the lung loss phenotype of Chinese Birt–Hogg–Dubé patients. The patient had a typical phenotype of BHD, including multiple papules visible on the skin of the head and neck, multiple pulmonary cysts with recurrent spontaneous pneumothorax, and a left renal cyst. The proband's Sister 1 had a similar clinical phenotype of multiple pulmonary cysts and renal cysts. The patient and Sister 1 were suspected of having BHD because of familial aggregation of symptoms of spontaneous emphysema and pulmonary cysts.

A gene test revealed a deletion mutation in the *FLCN* gene c.21_22del p. Cys8Profs⁣^∗^28, which was not available in the Leiden Open Variant Database and was not reported in the literature, making it a newly discovered *FLCN* mutation. The mutation probably originated in the mother, who had a history of recurrent pneumothorax and had died of cor pulmonale. To further clarify the diagnosis, based on the diagnostic criteria, this study designed a series of experiments to confirm whether the new *FLCN* gene mutation is pathogenic. The effect of *FLCN*-mut on *FLCN* gene expression was detected by transfecting *FLCN*-mut and *FLCN*-wt vectors, and the results showed that *FLCN* mutations significantly downregulate *FLCN* mRNA and protein levels. This suggested that novel mutations in *FLCN* may play a pathogenic role by reducing *FLCN* protein levels since the frameshift mutation gives rise to PTCs.

Since the sequencing data from this study confirmed the presence of a deletion mutation in *FLCN*, the authors hypothesized that the deletion mutation in *FLCN* may degrade the truncated protein through the NMD pathway, leading to the development of BHD. Therefore, an NMD assay was performed to verify the pathogenic mechanism of the c.21_22del frameshift mutation in Exon 4 of *FLCN*. The results illustrated that the administration of CHX+ or si-UPF1 did not significantly impact *FLCN* mRNA expression in the wild-type group but effectively mitigated the downregulation of *FLCN* mRNA expression in the mutant group. This suggested that CHX and si-UPF1 prevented the degradation of mutant *FLCN* mRNA through the inhibition of NMD. This may be related to the fact that the mutation was located in Exon 4, which was distant from the 5⁣′ proximal end and did not initiate NMD escape, and our bioinformatic prediction results (Figure S1) also suggested that the mutation site was in the NMD-competent region where NMD takes place. Since NMD experiments using the 293 T cell line cannot reflect the phenotypic characteristics of the patients, our study utilized further CRISPR/Cas9 to introduce the gene mutation in bronchial epithelial cells of human origin (BEAS-2B cells) associated with the *FLCN* lung phenotype, in order to simulate situations in vivo.

The knock-in of the *FLCN* mutation revealed similar results to those observed in NMD experiments in 293 T cells. In the present study, the NMD pathway degraded amounts of truncated proteins, resulting in haploinsufficiency that left patients with severe clinical symptoms. These findings provided further support for our hypothesis that NMD plays a role in the decreased expression of mutant *FLCN*.

To search for more pathogenic evidence according to ACMG guidelines, the effects of *FLCN* on other pathway proteins were analyzed by reviewing the literature. *FLCN* protein regulates cell growth, proliferation, and angiogenesis by inhibiting the mTOR pathways [26]. FLCN is a key molecule connecting energy-sensing signals to growth-suppressive TGF-*β* signaling [27]. *FLCN* is a critical regulator of the Wnt pathway via TFE3, and *FLCN*-dependent defects in Wnt pathway developmental cues may contribute to lung cyst pathogenesis in Birt–Hogg–Dubé [19]. Patients with Birt–Hogg–Dubé had a mutation in the *FLCN* gene, where the *FLCN* protein was incompetent. Further immunohistochemistry was performed to investigate the effects of gene mutations on the mTOR pathway, TGF-*β* pathway, and Wnt pathways in this patient.

The results showed that the positive rates of p-Smad3 and p-mTOR were higher in Birt–Hogg–Dubé patients compared with simple pulmonary bulla patients, and *β*-catenin was lower in Birt–Hogg–Dubé patients compared with simple pulmonary bulla patients. The expression level of p-mTOR is affected by *FLCN* mutations in BHD, which was related to the fact that mutant *FLCN* and its interacting partner, FNIP1, may be involved in energy and/or nutrient sensing through the AMPK and mTOR signaling pathways in BHD [28]. Mutant *FLCN* and its interacting partner, FNIP1, might be downstream effectors of mTOR and AMPK and modulate energy nutrient-sensing signaling pathways by some as-yet-unknown molecular mechanism. The expression level of TGF-*β* is affected by *FLCN* mutations in BHD, and the aberrant expression of TGF-*β* is closely associated with the development of a variety of tumors, suggesting that deregulation of genes involved in TGF-*β* signaling by *FLCN* mutation was likely to be an important step in the development of renal tumors in BHD [27].

Since *FLCN* is a critical regulator of the Wnt pathway via TFE3 [19], the lower *β*-catenin expression suggested that FLCN-dependent defects in Wnt pathway developmental cues may contribute to lung cyst pathogenesis in BHD. These results suggest that *FLCN* mutations can lead to BHD by regulating the mTOR pathway, TGF-*β* pathway, and Wnt pathway [29]. This may be one of the molecular mechanisms leading to BHD, but the exact mechanism needs to be further investigated.

According to “Criteria for classifying pathogenic variants” of ACMG standards and guidelines, the c.21_22del p.Cys8Profs⁣^∗^28 mutation meets one very strong evidence of pathogenicity (PSV1, the variant as a frameshift mutation, leading to premature termination of protein translation), one strong evidence (PS3, the mutation has been shown to result in decreased protein expression and impaired function by NMD assays and immunohistochemistry), one moderate evidence (PM2, the variant was not reported in the database and was rare mutation), one supporting evidence (PP1, mutation found in two symptomatic members of the family line, mutation cosegregation with disease) for *FLCN*, and one supporting evidence (PP3, multiple lines of computational evidence support deleterious effect on *FLCN*). Our study has verified that the mutation has a destructive effect on *FLCN* from the NMD assay. So it was reasonable to suggest that the c.21_22del p.Cys8Profs⁣^∗^28 mutation on Exon 4 of the *FLCN* gene was a kind of pathogenic mutation.

## 5. Conclusion

The present study detected a unique, novel *FLCN* frameshift mutation c.21_22del (p. Cys8 Profs⁣^∗^28) in Exon 4 from two members of a Chinese family highly suspected of BHD and summarized their clinical features. The study confirmed that *FLCN* mutations significantly downregulated both the mRNA and protein levels of FLCN. The truncated protein produced by the *FLCN* frameshift mutation was degraded via the NMD pathway. Expression of key proteins of the AMPK/mTOR signaling pathway by immunohistochemistry indicated that *FLCN* frameshift mutations were responsible for BHD through activation of the AMPK/mTOR signaling pathway. This study demonstrated that a novel *FLCN* frameshift mutation was responsible for the pathogenesis of BHD for the first time. It was also preliminarily demonstrated that *FLCN* causes BHD through the AMPK/mTOR signaling pathway. The specific mechanism awaits further confirmation and validation in subsequent studies.

## Figures and Tables

**Table 1:** Primer sequence for vector construction.

**Primer name**	**Primer sequence**
Phage-*FLCN*-BamHI-F	CGCTGGATCCATGAATGCCATCGTGGCTCT
Phage-*FLCN*-BamHI-mut-F	CGCTGGATCCATGAATGCCATCGTGGCTCTGCCACTTCTGCGAGCTCCAC
Phage-*FLCN*-NotI-R	CGACGCGGCCGCtGTTCCGAGACTCCGAGGCTG
pEGFP-C1-*FLCN*-BamHI-R	CGGTGGATCCTCAGTTCCGAGACTCCGAGG
*FLCN*-test-F	CGAGTTTGTGGTGACCAGTG
pEGFP-C1-*FLCN*-HindIII-F	GCTCAAGCTTacATGAATGCCATCGTGGCTCT
pEGFP-C1-*FLCN*-HindIII-mut-F	GCTCAAGCTTacATGAATGCCATCGTGGCTCTGCCACTTCTGCGAGCTCCAC

**Table 2:** Primer sequence for RT-qPCR.

**Primer name**	**Primer sequence**
*FLCN*-qPCR-F	GTCTGCCTCAAGGAGGAGTG
*FLCN*-qPCR-R	TAGGTCTTGCTCAGGCCAGT
UPF1-qPCR-F	AGAGGTGACCCTGCACAAGG
UPF1-qPCR-R	AGCCGAGGAGGAAGACGTTG

**Table 3:** Target sequence and primer sequence.

**Target**	**Target sequence**	**Primer sequence**
sgRNA1	GAAGTGGCAGAGAGCCACGA	F:CACCGAAGTGGCAGAGAGCCACGA
		R:AAACTCGTGGCTCTCTGCCACTTC
sgRNA2	TCCGTGCAGAAGAGAGTGCG	F:CACCgTCCGTGCAGAAGAGAGTGCG
		R:AAACCGCACTCTCTTCTGCACGGAC
sgRNA3	GGCCGTGGAGCTCGCAGAAG	F:CACCGGCCGTGGAGCTCGCAGAAG
		R:AAACCTTCTGCGAGCTCCACGGCC

**Table 4:** Primer sequence of donor vector construction.

**Primer name**	**Primer sequence**
8212-*FLCN*-F	GGCATGAGTGAACCTATTCAAG
8537-*FLCN*-F	CCTGCAGGAGGTAAGCTGCATG
*FLCN*-mut-F	TGCCATCGTGGCTCTGCCACTTCTGCGAGC
*FLCN*-mut-R	GCTCGCAGAAGTGGCAGAGCCACGATGGCA
9592-*FLCN*-F	GTGGCAGGCAAGTGTAATTCG
9904-*FLCN*-R	CAAGAGCTGATGGAGTGACCCAC

**Table 5:** Primer sequence for RT-qPCR.

**Primer name**	**Primer sequence**
*FLCN*-phage-QPCR-F	ATCCACGCTGTTTTGACCTC
*FLCN*-phage-QPCR-R	GACTGTCCTCATTCCCATCC
*FLCN*-qPCR-F	GTCTGCCTCAAGGAGGAGTG
*FLCN*-qPCR-R	TAGGTCTTGCTCAGGCCAGT
UPF1- qPCR-F	AGAGGTGACCCTGCACAAGG
UPF1- qPCR-R	AGCCGAGGAGGAAGACGTTG

## Data Availability

The datasets used and analyzed during the current study are available from the corresponding authors on reasonable request.

## Nomenclature

BHD: Birt–Hogg–Dubé
CHX: cycloheximide
DAB: diaminobenzidine
FBS: fetal bovine serum
*FLCN*: folliculin
NGS: next-generation sequencing
NMD: nonsense-mediated mRNA decay
PTC: premature termination codon
SD: standard deviation
VEP: variant effect predictor

## Supporting Information

Supporting Information Additional supporting information can be found online in the Supporting Information section. Figure S1: Prediction of whether variant leads to NMD. Our variant located at the NMD-competent region may lead to NMD. Figure S2: The design and validation of FLCN sgRNA and detection of FLCN mRNA expression by RT-qPCR. (A) The design of FLCN sgRNA. (B) The validation of FLCN sgRNA. (C) The detection of FLCN mRNA expression.⁣^∗∗^*p* < 0.01. Figure S3: Construction and verification of FLCN-KI monoclonal cell lines. (A) Culture of monoclonal cell lines. (B) Verification of FLCN-KI monoclonal cell line. (C) PCR products of positive cloned cell lines were connected to the T vector for sequencing.
