# Supplementary material for: Clinic Examination and Gene Diagnosis for a Birt–Hogg–Dubé Syndrome Family With a Novel flcn Frameshift Mutation Causing Nonsense-Mediated mRNA Degradation
Source: Hum Mutat. 2025 Feb 3;2025:7194418. doi: 10.1155/humu/7194418 (PMC12267975; doi:10.1155/humu/7194418)

Supplementary results


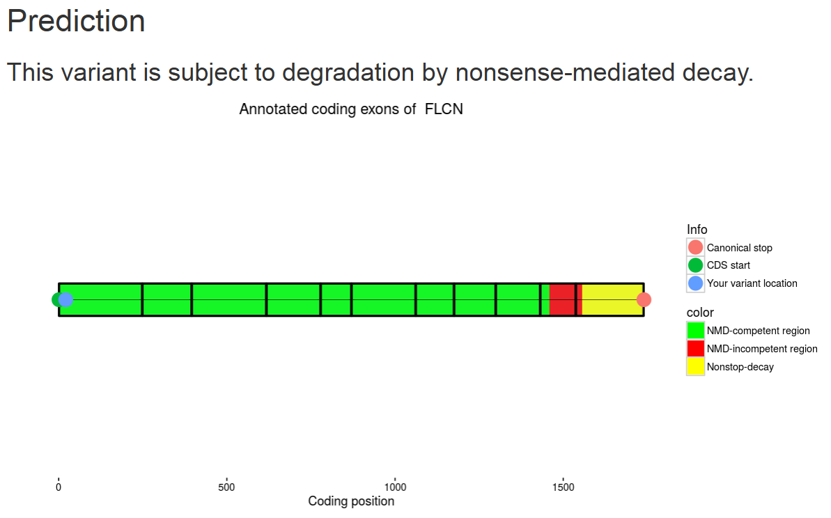


Supplementary Figure 1. Prediction of whether variant lead to NMD. Our variant located at NMD-competent region, may lead to NMD.


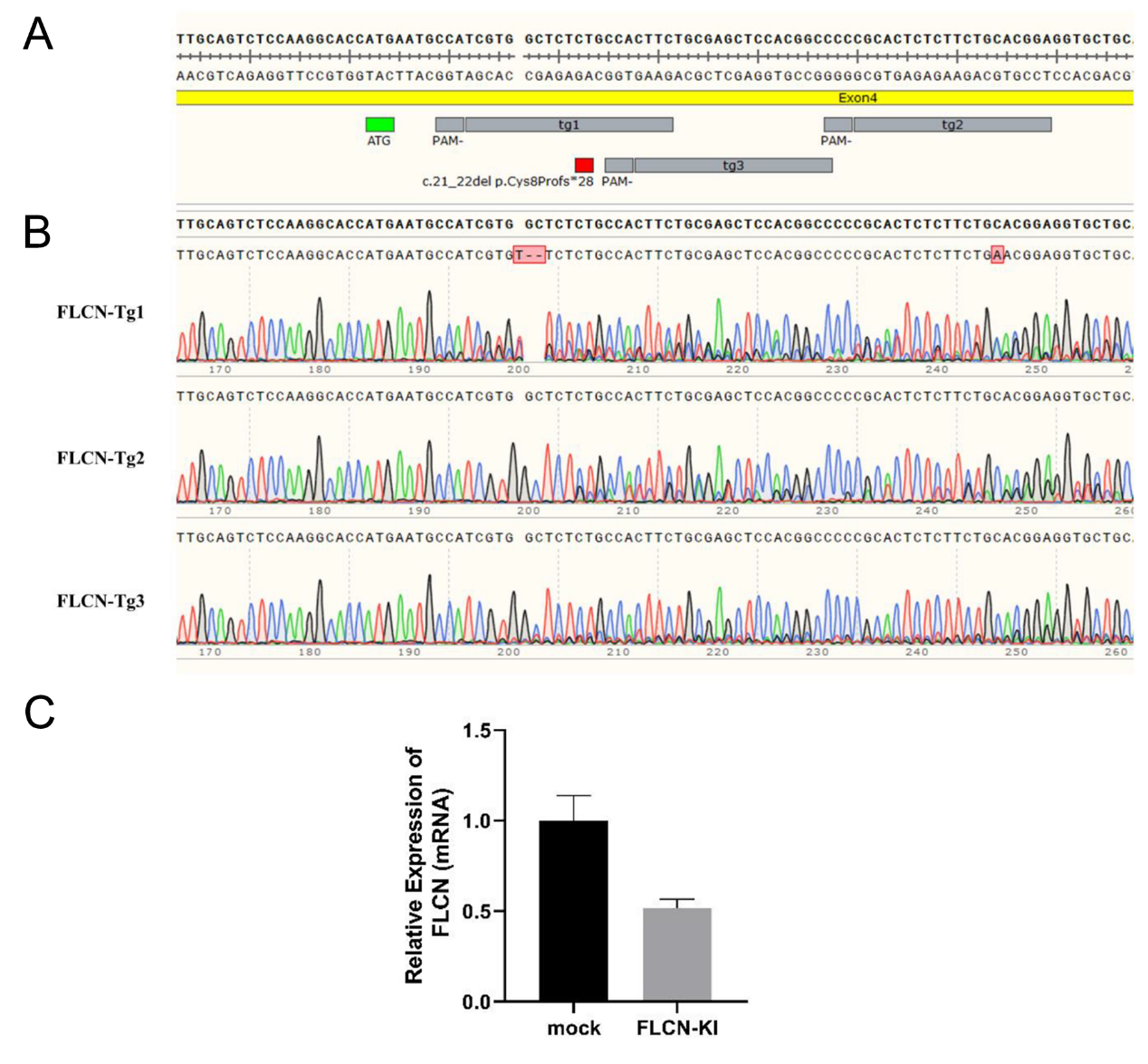
 Supplementary Figure 2. The design and validation of FLCN sgRNA and detection of FLCN mRNA expression by RT-qPCR. **A**: The design of FLCN sgRNA. **B**: The validation of FLCN sgRNA. **C**: detection of FLCN mRNA expression. ^**^*P*<0.01


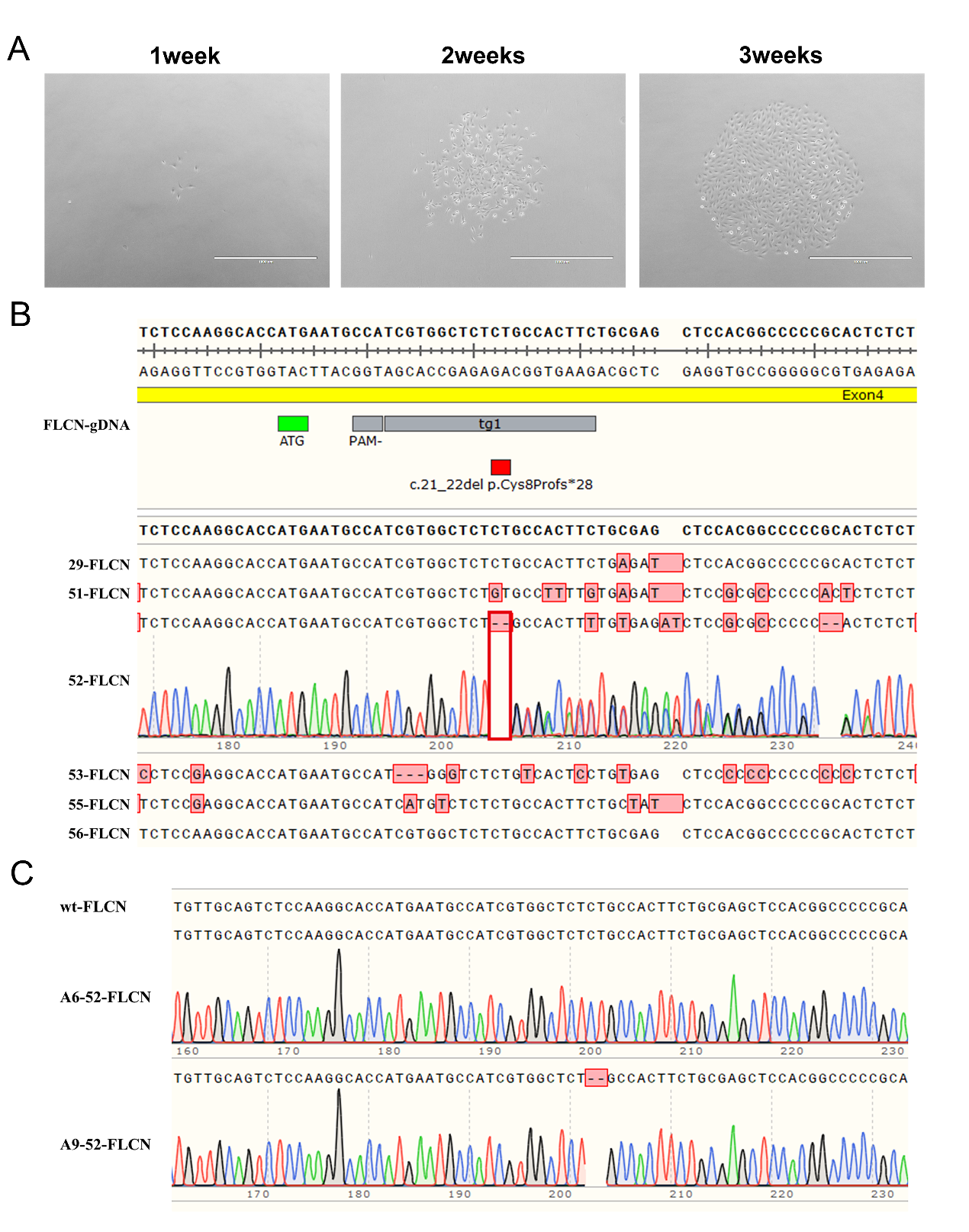
 Supplementary Figure 3. Construction and verification of FLCN-KI monoclonal cell lines. **A**: Culture of monoclonal cell lines. **B**: Verification of FLCN-KI monoclonal cell line. **C**: PCR products of positive cloned cell lines were connected to the T vector for sequencing.

3 repetitions of Figure 5C


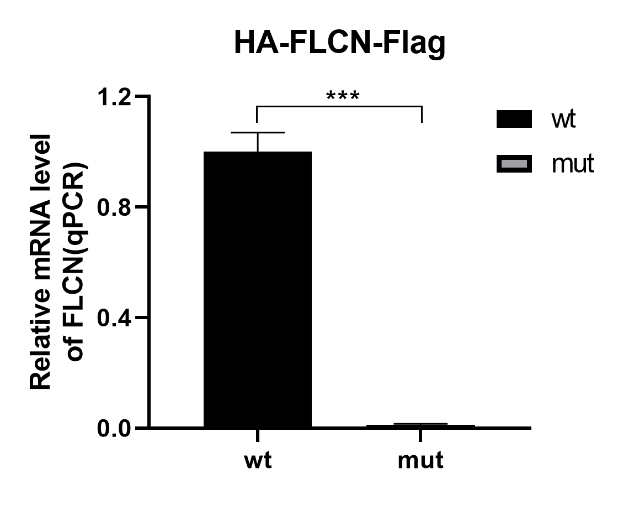

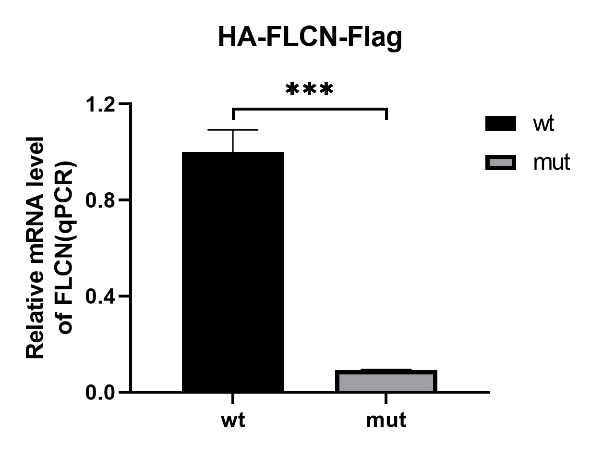


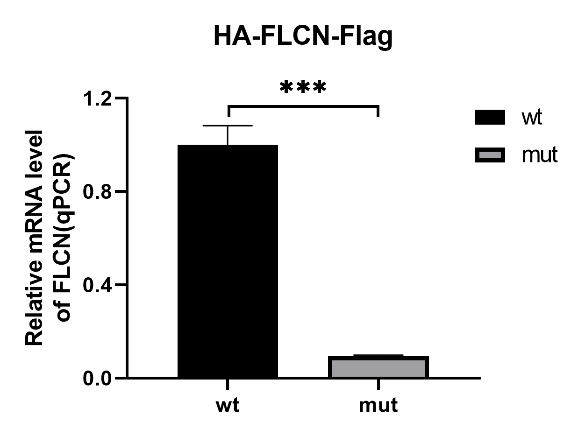


3 repetitions of Figure 5D


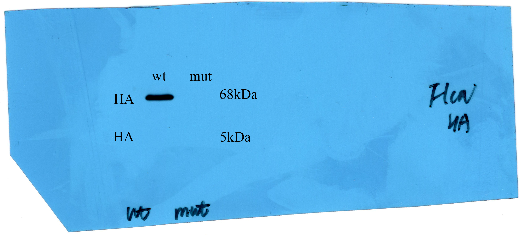

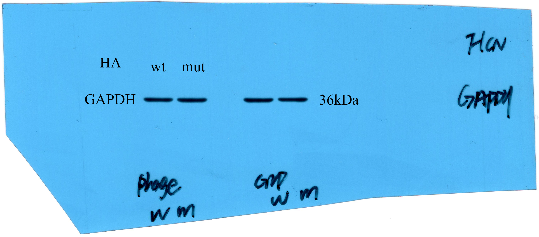


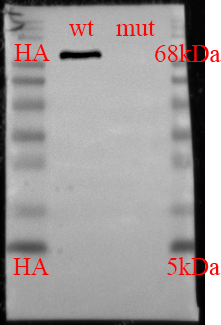

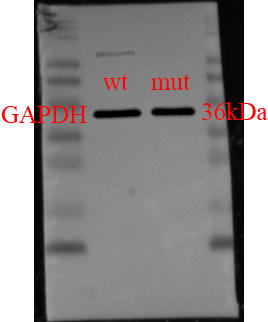


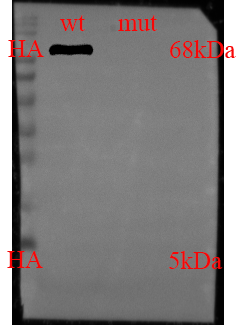

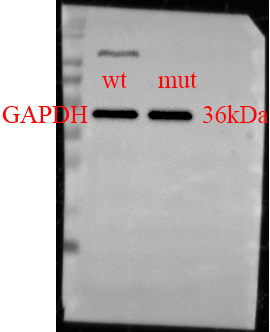


3 repetitions of Figure 6A


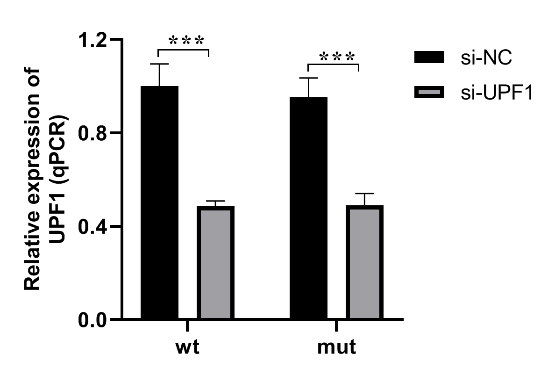

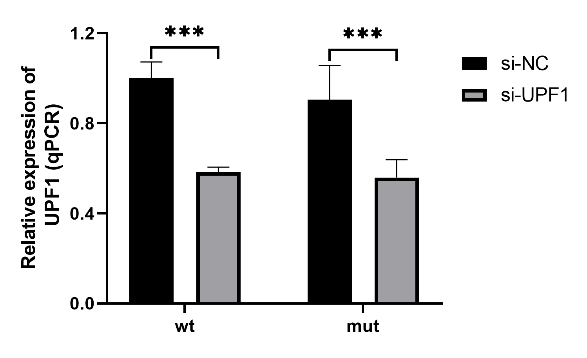


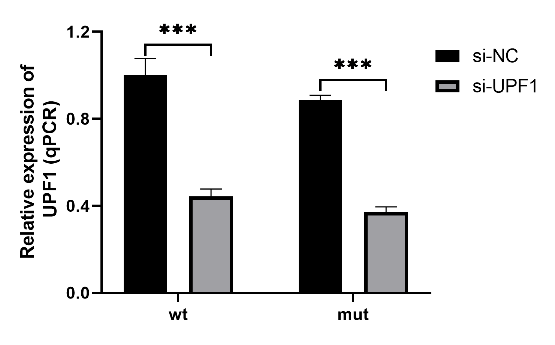


3 repetitions of Figure 6B


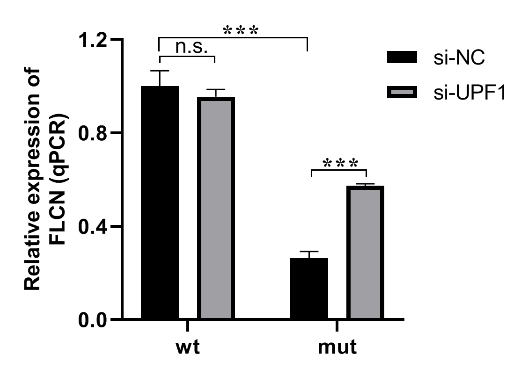

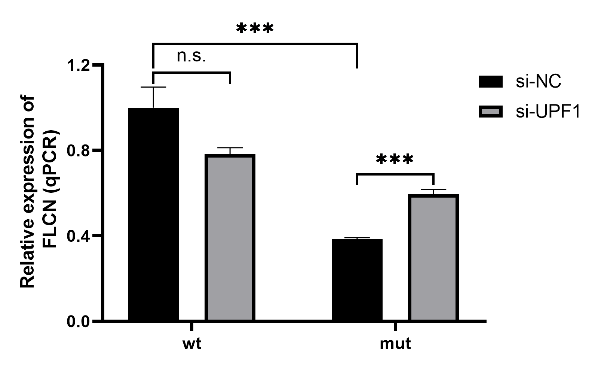


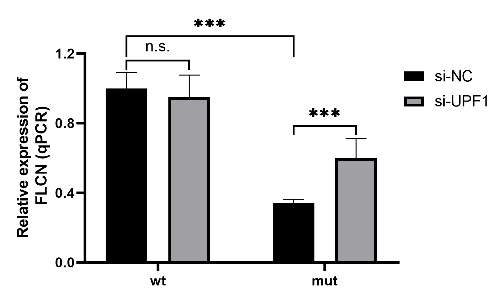


3 repetitions of Figure 6C


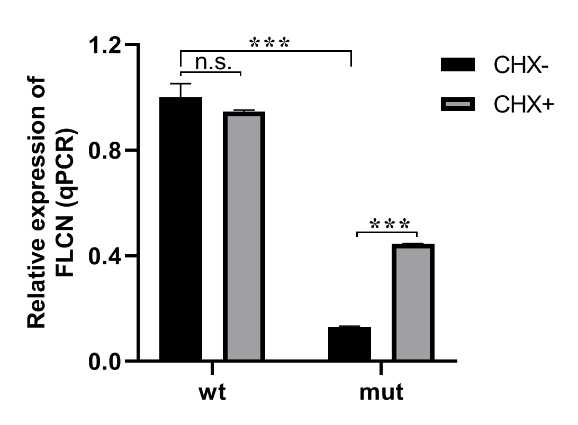

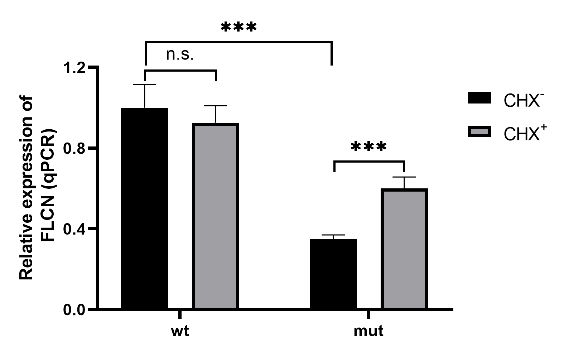


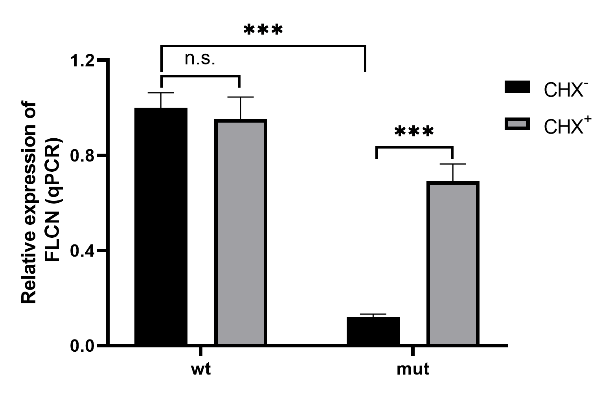


3 repetitions of Figure 6D


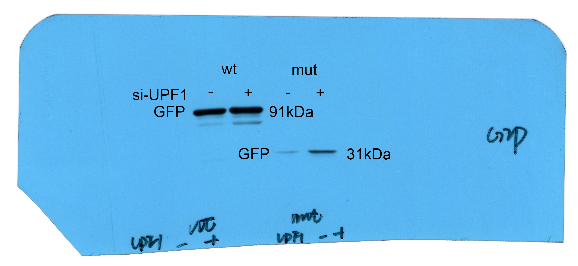

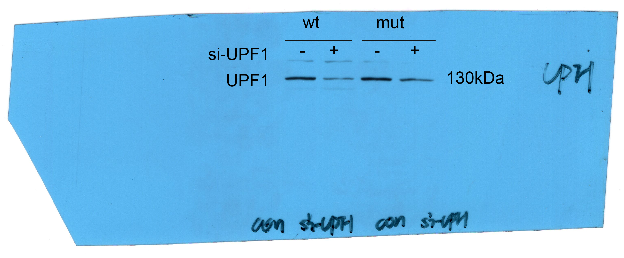


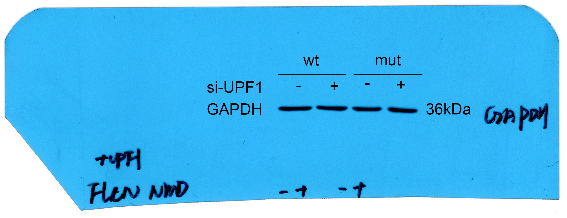


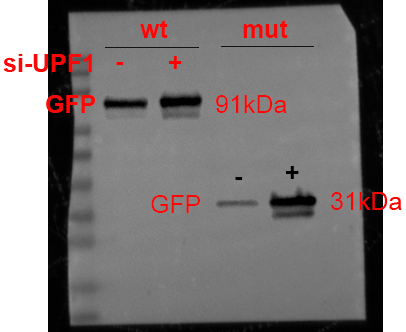

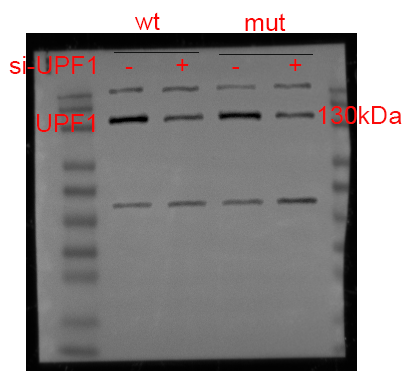


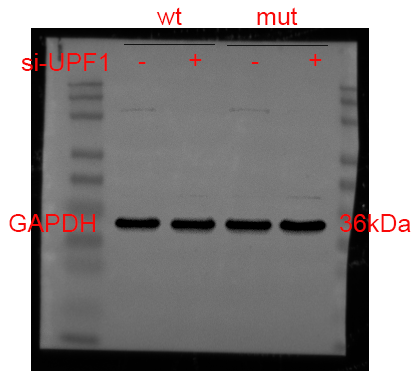


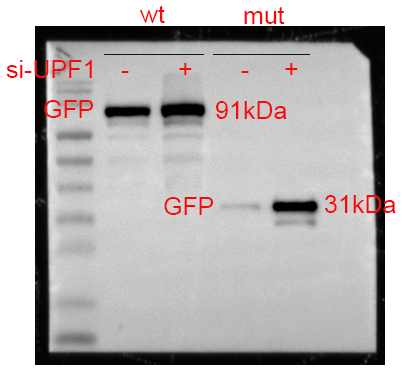

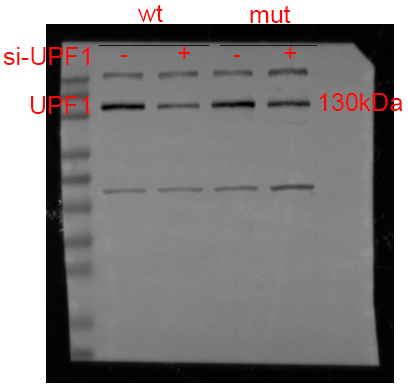


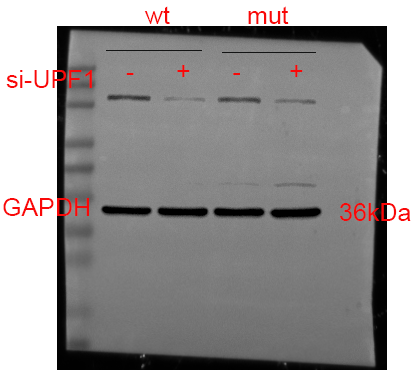


3 repetitions of Figure 7A


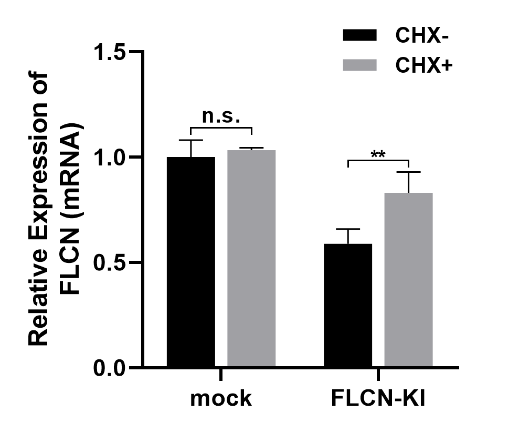

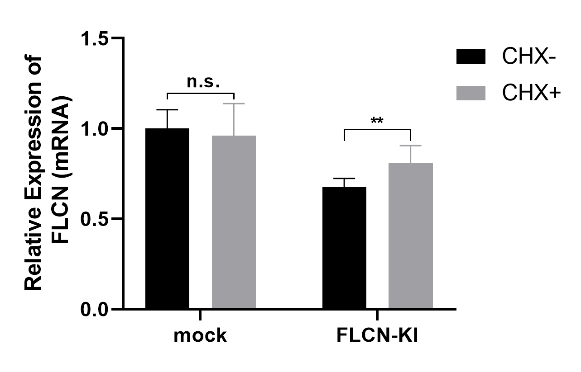


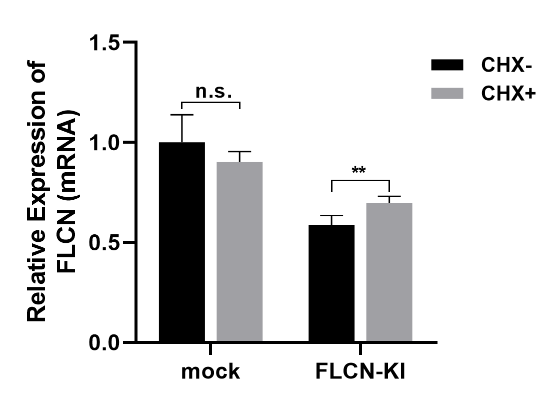


3 repetitions of Figure 7B


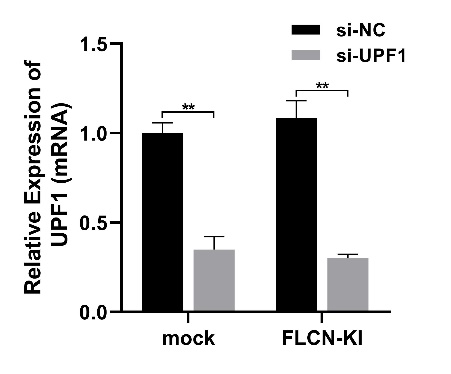

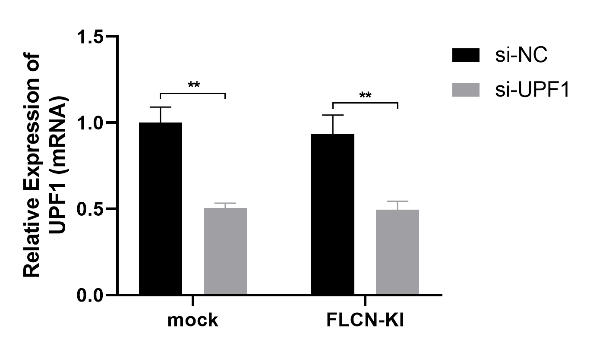

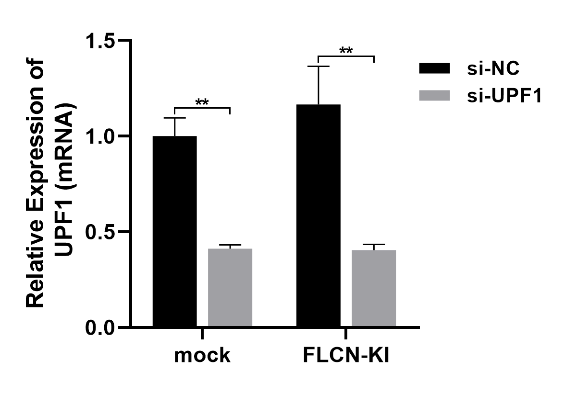


3 repetitions of Figure 7C


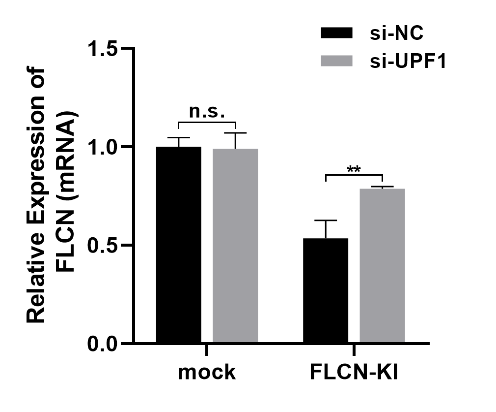

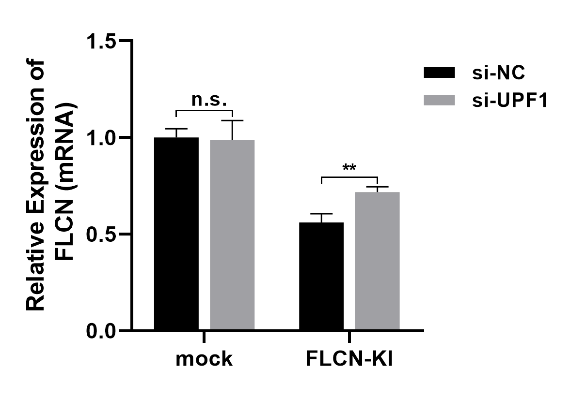


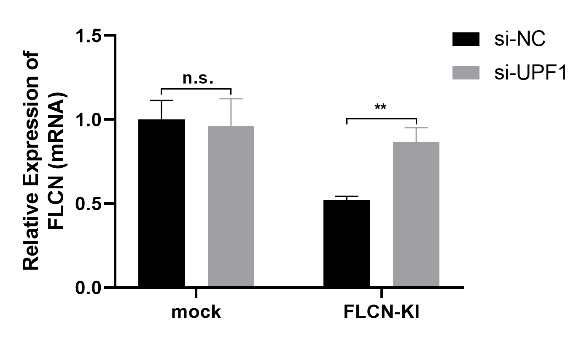

Supplement: Supporting Information — Additional supporting information can be found online in the Supporting Information section. Figure S1: Prediction of whether variant leads to NMD. Our variant located at the NMD-competent region may lead to NMD. Figure S2: The design and validation of FLCN sgRNA and detection of FLCN mRNA expression by RT-qPCR. (A) The design of FLCN sgRNA. (B) The validation of FLCN sgRNA. (C) The detection of FLCN mRNA expression.⁣∗∗p < 0.01. Figure S3: Construction and verification of FLCN-KI monoclonal cell lines. (A) Culture of monoclonal cell lines. (B) Verification of FLCN-KI monoclonal cell line. (C) PCR products of positive cloned cell lines were connected to the T vector for sequencing. [file 7194418.f1.docx]
